# Comparison of Bilateral and Unilateral Chronic Subdural Hematomas: A Retrospective Multi-institutional Cohort Study

**DOI:** 10.1227/neuprac.0000000000000261

**Published:** 2026-07-09

**Authors:** Anoop S. Chinthala, Trenton A. Line, Barnabas Obeng-Gyasi, Gordon Mao, Jamie Bradbury, Aditya Mittal, Ryan T. Kellogg, Enyinna Nwachuku, David O. Okonkwo, Jan Vargas, Matthew Pease

**Affiliations:** 1 Department of Neurological Surgery, Indiana University School of Medicine, Indianapolis, Indiana, USA;; 2 Department of Neurological Surgery, Allegheny Health Network, Pittsburgh, Pennsylvania, USA;; 3 Department of Neurological Surgery, Indiana University Health, Indianapolis, Indiana, USA;; 4 Department of Neurological Surgery, University of Utah Health, Salt Lake City, Utah, USA;; 5 Department of Neurosurgery, University of Virginia, Charlottesville, Virginia, USA;; 6 Department of Neurosurgery, Penn State Health, Hershey, Pennsylvania, USA;; 7 Department of Neurosurgery, UPMC Healthcare System, Pittsburgh, Pennsylvania, USA;; 8 Division of Neurosurgery, PRISMA Health, Greenville, South Carolina, USA

**Keywords:** Chronic subdural hematoma, Unilateral, Bilateral, Recurrence

## Abstract

**BACKGROUND AND OBJECTIVES:**

Chronic subdural hematoma (cSDH) is a major public health issue in the United States. cSDH can present as unilateral or bilateral collections. We compared outcomes of surgically treated unilateral and bilateral cSDH and assessed factors contributing to retreatment.

**METHODS:**

We performed a retrospective cohort study through chart review of patients operated for cSDH from 4 level 1 trauma centers, February 2009 to August 2021. Patients were categorized into 3 groups: (1) unilateral, operated cSDH; (2) bilateral, operated cSDH on both sides; and (3) bilateral cSDH, but only one side operated. Patients with middle meningeal artery embolization were excluded. Data on demographics, clinical presentation, radiological findings, and outcomes were collected. A multivariable logistic regression model assessed factors associated with retreatment.

**RESULTS:**

We analyzed a total of 731 patients: 563 (77%) had unilateral operated cSDH; 132 (18%) had bilateral operated cSDH; and 36 (5%) had bilateral cSDH but were operated only on one side. We found no significant differences in retreatment rates between patients with bilateral, operated cSDH and those with unilateral, operated cSDH (14% vs 12%, *P* = .40), 30-day readmission (30% vs 25%, *P* = .20), or 30-day mortality (5.3% vs 8.0%, *P* = .30). Increased postoperative volume was associated with a higher risk of retreatment (odds ratio 3.24, 95% CI 2.15-5.07, *P* < .001). Compared with those with unilateral, operated cSDH, patients with bilateral cSDH operated on one side showed no significant difference in retreatment (11% vs 12%, *P* > .9), 30-day readmission (14% vs 25%, *P* = .12), or 30-day mortality (5.3% vs 8.0%, *P* > .9).

**CONCLUSION:**

There was no significant difference in retreatment, 30-day readmission, or 30-day mortality between unilateral and bilateral cSDHs treated surgically. Despite exclusion of bilateral cSDH from major recent clinical trials, their rates of recurrence are similar to unilateral cSDH.

**ABBREVIATIONS:**
cSDH: chronic subdural hematoma
CT: computed tomography
EG: esophagogastric
GCS: Glasgow Coma Scale
MMA: middle meningeal artery

As the population ages, chronic subdural hematoma (cSDH) is increasing in frequency and is projected to become the most common condition requiring neurosurgical intervention. The annual incidence ranges widely from 2 to 80 per 100 000 people but rises sharply in the elderly, with rates approaching 130 per 100 000 among those older than 80 years.^1-3^ This increased vulnerability stems from a combination of age-related cerebral atrophy and the prevalent use of anticoagulant and antiplatelet medications in older adults, which increases hemorrhage rates even after minor head trauma.^4^ As recurrence remains common and treatment burdens persist, cSDH poses a challenge, not only in clinical management but also in healthcare utilization and cost.^5^

cSDH can present as either unilateral or bilateral collections, with bilateral cSDH accounting for approximately 14% to 25% of all cases.^6^ Emerging literature suggests that bilateral cSDH may represent a distinct pathology compared with unilateral cSDH, with reports of worse clinical presentations, radiological findings, and outcomes.^7-10^ Bilateral cSDH is reported to present with worse mental status and neurological signs at both baseline and postoperative state.^11^ However, these data have been inconclusive, as other studies have suggested bilateral cSDH have favorable postoperative outcomes and do not significantly differ in neurological presentation to unilateral cSDH.^11,12^ Recent, large-scale randomized clinical trials have excluded bilateral cSDH from outcome analyses, limiting our understanding of treatment in this patient population.^13-18^

Owing to these conflicting findings, outcomes for bilateral cSDH remain unclear. Retreatment for bilateral cSDH is suggested to occur at a higher rate than unilateral cSDH and to be an independent risk factor of recurrence; however, these studies include only a small sample size of bilateral cSDH for analysis.^9,19,20^ We aimed to evaluate differences in clinical presentation, radiographic features, and recurrence rates between bilateral and unilateral cSDH by analyzing a large, multicenter US cohort of bilateral cSDH cases. This analysis provides an opportunity to better characterize the unique pathology of bilateral cSDH and to identify distinctions that may inform treatment planning and prognostic assessment.

## METHODS

The study was conducted in accordance with the Declaration of Helsinki and the Human Research Protection Office at all sites approved this study without need for consent.

### Patient Selection

We identified patients between February 2009 to August 2021 from 4 level 1 trauma centers affiliated with 3 institutions using Common Procedural Technology codes for craniotomy and burr holes and/or craniotomy for subdural hematoma evacuation. Exclusion criteria included patients who had acute subdural hematomas, previous neurosurgical pathology, underwent preoperation or postoperation middle meningeal artery (MMA) embolization, uninterpretable admission or postoperative nonconstrasted head computed tomography (CT) scans, or bilateral cSDH with a minor side volume <5 mL. We retrospectively reviewed patient records to gather demographic details, baseline clinical information, and outcome data. Outcomes of interest included retreatment, defined as subsequent surgical treatment, excluding infections, within 6 months of index surgery, 30-day readmission, and 30-day mortality. All postoperative head CTs were obtained within 48 hours of surgery.

Across all 3 institutions, treatment practices for cSDH were consistent. Neurosurgeons elected to operate on patients with large hematomas, significant midline shift, or concerning symptoms. Surgeons performed hematoma evacuation using either burr holes or mini-craniotomy, depending on clinical judgment. MMA embolization was not widely used at these institutions during the data collection phase as this took place in a time before it became common practice. They routinely placed subdural or subgaleal drains postoperatively and typically removed them within 2 to 3 days.^21^ To estimate hematoma volumes, we applied a previously validated 3-dimensional deep learning model based on convolutional neural networks. This model was trained on over 21 000 manually labeled images from 128 head CT scans, which included both preoperative and postoperative studies from patients treated surgically for cSDH. We processed CTs using a soft tissue reconstruction algorithm to enhance contrast between intracranial structures. Coronal images were prioritized for segmentation due to superior visualization of hematoma boundaries; axial views were used when coronal reconstructions were unavailable. Automated segmentation outputs were then used to derive hematoma volume measurements.^22^

### Statistical Analysis

We analyzed baseline clinical characteristics and outcomes in patients with cSDHs by grouping them based on hematoma laterality and surgical treatment. We divided patients into 3 categories: (1) unilateral, operated cSDH; (2) bilateral, operated cSDH on both sides; and (3) bilateral cSDH, but only one side operated. This classification allowed us to systematically compare clinical features and outcomes across distinct treatment patterns.

We compared continuous variables between groups using the Wilcoxon rank-sum test and assessed categorical variables using either the χ^2^ test or Fisher exact test, depending on sample size and distribution.

To evaluate our primary outcome of interest—retreatment—a multivariable logistic regression model comparing surgically treated unilateral and bilateral cSDH patients examined the association between hematoma laterality and the likelihood of repeat surgery. The Box-Tidwell test confirmed the linearity assumption for continuous variables and variance inflation factors assessed multicollinearity. Predictor variables were selected based on prior work from our group evaluating retreatment predictors in unilateral cSDH, in which candidate variables including radiographic features and clinical factors were assessed and reduced using bidirectional stepwise selection based on Akaike Information Criterion.^23^ All analyses were performed using RStudio 2024.09.0+375 (RStudio, PBC).

## RESULTS

We identified 738 patients surgically treated for cSDH from February 2009 to August 2021. After excluding patients for MMA embolization (2) and bilateral cSDH with a minor side volume <5 mL (5), we included 731 patients for analysis. Of these, 563 had unilateral operated cSDH, 132 had bilateral operated cSDH, and 36 had bilateral cSDH but were operated on one side. Characteristics of all 3 groups can be found in **Supplemental Digital Content 1** (**Table**, http://links.lww.com/NS9/A116).

### Comparison of Bilateral, Operated cSDH on Both Sides and Unilateral, Operated CSDH

Table 1 summarizes the clinical characteristics of patients with bilateral cSDH operated on both sides vs those with unilateral cSDH. Admission Glasgow Coma Scale (GCS) scores were also comparable, with no significant differences (*P* = .2). Anticoagulation use was significantly more common in the bilateral group (34% vs 15%, *P* = .0002). The bilateral group also had smaller mean midline shift (3.6 ± 3.5 mm vs 8.5 ± 4.5 mm, *P* < .001). Preoperative and postoperative hematoma volumes were significantly larger in the bilateral group (preoperative: 170 ± 58 mL vs 108 ± 42 mL; postoperative: 74 ± 45 mL vs 47 ± 31 mL; both *P* < .001). Age, gender, admission GCS, and antiplatelet use did not differ significantly.

**TABLE 1.** Demographic and Clinical Characteristics of Bilateral cSDH Operated on Both Sides and Unilateral, Operated cSDH

Variable	Bilateral cSDH (operated on both sides) N = 132	Unilateral cSDH N = 563	*P*-value
Age (y)	74 ± 10	72 ± 13	.2
Sex (%)			.12
Female	31 (23)	171 (30)	
Male	101 (77)	392 (70)	
Admission GCS	14.38 ± 1.43	14.26 ± 1.57	.2
Anticoagulation (%)	34 (26)	82 (15)	.002^a^
Antiplatelet (%)	51 (39)	260 (46)	.12
Admission platelet count (U/mL)	229 ± 92	234 ± 92	.4
Seizure (%)	9 (6.8)	24 (8.3)	.6
Midline shift (mm)	3.6 ± 3.5	8.5 ± 4.5	<.001^a^
Preoperative cSDH volume (mL)	170 ± 58	108 ± 42	<.001^a^
Postoperative cSDH volume (mL)	74 ± 45	47 ± 31	<.001^a^
Length of stay (d)	7.3 ± 5.2	7.3 ± 7.0	.069
Retreatment (%)	19 (14)	65 (12)	.4
30-d readmission (%)	40 (30)	143 (25)	.2
30-d mortality (%)	7 (5.3)	45 (8.0)	.3

cSDH, chronic subdural hematoma; GCS, Glasgow Coma Scale.

a Statistically significant values.

Categorical variables are displayed as n (%); continuous variables are displayed as mean ± SD.

We found no significant differences in retreatment rates between patients with bilateral, operated cSDH and those with unilateral, operated cSDH (14% vs 12%, *P* = .40), 30-day readmission (30% vs 25%, *P* = .20), or 30-day mortality (5.3% vs 8.0%, *P* = .30).

### Comparison of Bilateral cSDH Operated on One Side and Unilateral, Operated cSDH

Table 2 compares patients with bilateral cSDH operated on one side to those with unilateral, operated cSDH. Burr hole drainage was more frequently used in the bilateral one-sided group (83% vs 52%, *P* < .001). Preoperative (75 ± 57 mL) and postoperative (91 ± 41 mL) volumes were significantly larger in the bilateral group (both *P* < .001). Midline shift was smaller in the bilateral group (5.9 ± 3.1 mm vs 8.5 ± 4.5 mm, *P* = .007). The groups showed no significant difference in age or admission GCS, although the unilateral group included more females (*P* = .035).

**TABLE 2.** Demographic and Clinical Characteristics of Bilateral cSDH Operated on One Side and Unilateral, Operated cSDH

Variable	Bilateral cSDH (operated on one side) N = 36	Unilateral cSDH N = 563	*P*-value
Age (y)	71 ± 14	72 ± 13	.5
Gender (%)			.035^a^
Female	5 (14)	171 (30)	
Male	31 (86)	392 (70)	
Admission GCS	13.55 ± 2.65	14.26 ± 1.57	.3
Anticoagulation (%)	10 (28)	82 (15)	.033^a^
Antiplatelet (%)	22 (61)	260 (46)	.082
Admission platelet count (U/mL)	222 ± 53	234 ± 92	.8
Seizure (%)	3 (8.3)	24 (8.3)	>.9
Midline shift (mm)	6.0 ± 3.1	8.5 ± 4.5	.007^a^
Preoperative cSDH volume (mL)	156 ± 57	108 ± 42	<.001^a^
Surgical treatment (%)			<.001^a^
Treated with burr hole	30 (83)	292 (52)	
Treated with craniotomy	6 (17)	271 (48)	
Postoperative cSDH volume (mL)	91 ± 41	47 ± 31	<.001^a^
Length of stay (d)	15.4 ± 40.8	7.3 ± 7.0	.2
Retreatment (%)	4 (11)	65 (12)	>.9
30-d readmission (%)	5 (14)	143 (25)	.12
30-d mortality (%)	3 (8.3)	45 (8.0)	>.9

cSDH, chronic subdural hematoma; GCS, Glasgow Coma Scale.

a Statistically significant values.

Categorical variables are displayed as n (%); continuous variables are displayed as mean ± SD.

Patients with bilateral cSDH operated on one side showed no significant difference in retreatment (11% vs 12%, *P* > .9), 30-day readmission (14% vs 25%, *P* = .12), or 30-day mortality (5.3% vs 8.0%, *P* > .9) compared with those with unilateral, operated cSDH.

### Multivariable Analysis of Factors Associated with Retreatment

A multivariable logistic regression model assessed predictors of retreatment, including cSDH laterality, postoperative hematoma volume, and anticoagulation use. These selected variables have been previously identified to be significant predictors for cSDH retreatment.^23^ Patients with bilateral cSDH treated on only one side (N = 36) were excluded, resulting in a final sample of 695 patients. Log transformation of postoperative volume was performed to meet the linearity assumption. All variables met criteria for inclusion, with no evidence of multicollinearity.

Log-transformed postoperative cSDH volume significantly predicted retreatment (odds ratio [OR] 3.24, 95% CI 2.15-5.07, *P* < .001). Anticoagulation use (OR 1.64, 95% CI 0.90-3.87, *P* = .094) and bilateral cSDH (OR 0.74, 95% CI 0.40-1.31, *P* = .317) were not significant predictors.

## DISCUSSION

In this retrospective, multicenter cohort study, operatively treated bilateral and unilateral chronic SDH collections showed no difference in risk for retreatment, 30-day readmission, or 30-day mortality. The only significant factor identified by our regression model was postoperative cSDH volume, with increased postoperative volume corresponding to approximately triple the risk of retreatment (Table 3). Postoperative volume may represent a surrogate marker of surgical adequacy, residual hematoma burden, or perioperative management factors rather than a purely independent biological predictor of recurrence. Larger residual collections after surgery may reflect incomplete evacuation, differences in surgical technique, or patient-specific factors influencing hematoma clearance. Our results challenge the notion that bilateral cSDH inherently differs from unilateral cSDH in terms of retreatment, readmission, and mortality.^7-10,19,20^ However, these findings should be interpreted within the context of patients selected for surgical treatment under current clinical practice patterns. Comparisons between unilateral and bilateral cSDH may reflect differences in disease presentation and treatment selection rather than intrinsic biological equivalence between the 2 entities.

**TABLE 3.** Multivariable Logistic Regression Results

Variable	Odds ratio	Lower 95% CI	Upper 95% CI	*P*-value
Bilateral cSDH	0.739	0.400	1.313	.317
Postoperative cSDH volume (log-transformed)	3.239	2.147	5.065	<.001
Anticoagulation use	1.637	0.902	2.870	.094

cSDH, chronic subdural hematoma.

The population of bilateral cSDH is not well studied compared with unilateral cSDH. While earlier single-institution studies, often limited by small sample sizes, reported higher recurrence rates in bilateral cSDH,^9,24-26^ more recent analyses have found no significant difference,^11,27^ consistent with our findings. Many recent, large multicenter studies excluded bilateral cSDH, limiting our understanding of treatment for bilateral cSDH.^13-18^ Our results suggest that hematoma laterality alone does not determine prognosis. Other clinical and radiographic features appear to play a more influential role. Management decisions should therefore remain guided by individualized clinical and imaging findings, as is standard practice for unilateral cSDH.

Univariate analysis revealed no significant differences in age, admission GCS, preoperative antiplatelet use, or admission platelet count between operatively treated bilateral cSDH and unilateral cSDH. Although cSDH commonly affects older adults, and some have proposed a higher likelihood of bilateral cSDH in older patients,^12,28^ our study found no age-related differences between the groups. Regarding radiological parameters, bilateral cSDH were found to have less midline shift and higher prepreparative and postpreparative volume than unilateral cSDH, perhaps an expected finding.

Anticoagulant and antiplatelet use have been previously identified as risk factors for cSDH, particularly for bilateral cSDH.^12,29-31^ Anticoagulation use has also been associated with an increased risk of recurrence.^29^ Consistent with this, both bilateral cSDH cohorts—operated on both sides or just one side—showed higher anticoagulant use compared with unilateral cSDH. While anticoagulation use has been shown to contribute to recurrence in earlier studies, our findings indicate that when postoperative volume is accounted for, anticoagulation use is not a significant predictor.^31,32^ This likely reflects robust practices reversing anticoagulation. It is important to note that earlier studies focused on unilateral cSDH only, while our study extends these findings to include bilateral cSDH patients.

Further stratification demonstrated no significant differences in recurrence, 30-day readmission, and 30-day mortality between bilateral cSDH operated on one side and unilateral, operated cSDH (Table 2). This may suggest that, depending on radiological findings, treating only the dominant side in bilateral cases could be a viable approach in certain cases. Previous studies have yielded mixed results on unilateral vs bilateral decompression in bilateral cSDH, with some reporting an increased retreatment risk from unilateral decompression, and others showing no difference.^27,33^ Our small sample of bilateral cSDH patients treated on the dominant side (n = 36) represents a highly selected and clinically heterogeneous population and limits broader comparisons, highlighting the need for larger studies to further explore optimal management strategies for bilateral cSDH.

### Limitations

The retrospective design relies on data extracted from medical records, introducing the potential for both selection and information bias. The accuracy and completeness of documentation may vary across institutions and providers, which could influence data quality, particularly for clinical variables not routinely recorded. Treatment decisions, such as laterality of surgery or timing of intervention, were made at the discretion of the attending neurosurgeon, introducing variability in practice patterns that may affect outcomes. Comparisons between unilateral and bilateral cSDH may be influenced by selection bias inherent to surgical decision-making. As such, the bilateral and unilateral cohorts may represent distinct disease presentations rather than identical pathological entities. Although our regression model incorporated predictors previously identified in prior work, other potential confounders, including surgical technique, drain management strategies, institutional variation, were not included in the final model and may contribute to residual confounding. The study period (2009-2021) largely preceded the widespread adoption of MMA embolization for cSDH. Although patients who underwent MMA embolization were excluded from our analysis, the increasing use of adjunctive MMA embolization in modern practice may alter recurrence rates and retreatment patterns. Future studies evaluating cohorts treated in the era of routine MMA embolization will be important to determine whether these findings remain applicable within contemporary treatment paradigms.

## CONCLUSION

This multicenter, retrospective cohort study compared outcomes of surgically treated unilateral and bilateral cSDH. Hematoma laterality was not a significant predictor of retreatment; only postoperative volume was associated with increased risk. Outcomes including retreatment, 30-day readmission, and 30-day mortality were similar across groups, even when stratifying bilateral cases by extent of surgery. These findings suggest that bilateral cSDH does not inherently confer a worse prognosis and surgical decision-making should be on individual clinical and radiographic factors.

## Supplementary Material

## Supplemental Digital Content

**Supplemental Digital Content 1: Table.** Baseline characteristics and outcomes of patients with unilateral cSDH, bilateral cSDH treated on one side, and bilateral cSDH treated on both sides. cSDH, chronic subdural hematoma; GCS, Glasgow Coma Scale. Categorical variables are displayed as n (%); Continuous variables are displayed as mean ± SD Pearson's χ^2^ test, Kruskal-Wallis rank sum test, and Fisher's exact test were used as appropriate based on variable type and distribution. *Statistically significant values.

## COMMENTS

The authors are to be commended for assembling this large, multicenter dataset evaluating outcomes of surgically treated unilateral vs bilateral chronic subdural hematoma (cSDH). The dataset is substantial, and the topic is clinically relevant. The authors highlight that, in their cohort, postoperative volume was the only factor associated with increased risk of retreatment. Bilaterality itself was not associated with retreatment, 30-day readmission, or 30-day mortality. This is clinically reassuring and pushes back on the lingering perception that bilateral cSDH carries a categorically worse short-term operative prognosis.

This work opens 3 threads which merit continued dialogue and future investigation. First, postoperative residual volume remains analytically vexing. Even reframed as a marker of surgical adequacy, it intertwines perioperative decision making including surgical technique, drain placement, and physiologic factors such as comorbidity and brain re-expansion dynamics. Future work might prespecify it as a surgical-quality covariate rather than an independent prognostic variable. Second, the chosen endpoints capture early catastrophic failure but miss the postacute and long-term recovery that patients and families value most. Functional status, cognition, and return-to-baseline activity are conspicuously underrepresented in the cSDH outcomes literature generally. Third, this analysis arrives as middle meningeal artery embolization reshapes the treatment algorithm, particularly for bilateral disease, where staged or adjunctive endovascular management is increasingly attractive. The present data establish a useful surgical baseline against which embolization-era cohorts can be compared.

Ultimately, no retrospective dataset can fully adjudicate selection effects. A prospective registry or pragmatic trial stratified by laterality and adjunctive embolization is the natural next step. In the meantime, the authors give clinicians reasonable grounds to approach bilateral cSDH as a surgically manageable condition rather than an automatic marker of poor prognosis.

**John Harold Kanter**

*Pittsburgh, Pennsylvania, USA*

## References

[1] Feghali J Yang W Huang J. Updates in chronic subdural hematoma: epidemiology, etiology, pathogenesis, treatment, and outcome. World Neurosurg. 2020;141:339-345. 32593768 10.1016/j.wneu.2020.06.140

[2] Karibe H Kameyama M Kawase M Hirano T Kawaguchi T Tominaga T. Epidemiology of chronic subdural hematoma. Neurol Surg. 2011;39(12):205-210. 22128269

[3] Baiser D Farooq S Mehmood T Reyes M Samadani U. Actual and projected incidence rates for chronic subdural hematomas in United States Veterans Administration and civilian populations. J Neurosurg. 2015;123(5):1209-1215. 25794342 10.3171/2014.9.JNS141550 PMC4575892

[4] Rust T Kiemer N Erasmus A. Chronic subdural haematomas and anticoagulation or anti-thrombotic therapy. J Clin Neurosci. 2006;13(8):823-827. 16997707 10.1016/j.jocn.2004.12.013

[5] Rauhala M Helén P Huhtala H Chronic subdural hematoma-incidence, complications, and financial impact. Acta Neurochir (Wien). 2020;162(9):2033-2043. 32524244 10.1007/s00701-020-04398-3 PMC7415035

[6] Park HS Park ES Park JB Chronic subdural hematomas: comparison between unilateral and bilateral involvement. Korean J Neurotrauma. 2014;10(2):55-59. 27169034 10.13004/kjnt.2014.10.2.55 PMC4852612

[7] Lee J Park JH. Clinical characteristics of bilateral versus unilateral chronic subdural hematoma. Korean J Neurotrauma. 2014;10(2):49-54. 27169033 10.13004/kjnt.2014.10.2.49 PMC4852599

[8] Agawa Y Mineharu Y Tani S Adachi H Imamura H Sakai N. Bilateral chronic subdural hematoma is associated with rapid progression and poor clinical outcome. Neurol Med Chir (Tokyo). 2016;56(4):198-203. 26923835 10.2176/nmc.oa.2015-0256 PMC4831946

[9] Huang YH Yang KY Lee TC Liao CC. Bilateral chronic subdural hematoma: what is the clinical significance? Int J Surg. 2013;11(7):544-548. 23707986 10.1016/j.ijsu.2013.05.007

[10] Kurokawa Y Ishizaki E Inaba KI. Bilateral chronic subdural hematoma cases showing rapid and progressive aggravation. Surg Neurol. 2005;64(5):444-449. 16253697 10.1016/j.surneu.2004.12.030

[11] Hsieh CT Su IC Hsu SK Huang CT Lian FJ Chang CJ. Chronic subdural hematoma: differences between unilateral and bilateral occurrence. J Clin Neurosci. 2016;34(12):252-258. 27742369 10.1016/j.jocn.2016.09.015

[12] Tsai TH Lieu AS Hwang SL Huang TY Hwang YF. A comparative study of the patients with bilateral or unilateral chronic subdural hematoma: precipitating factors and postoperative outcomes. J Trauma. 2010;68(3):571-575. 20065879 10.1097/TA.0b013e3181a5f31c

[13] Hutchinson PJ Edlmann E Bulters D Trial of dexamethasone for chronic subdural hematoma. N Engl J Med. 2020;383(27):2616-2627. 33326713 10.1056/NEJMoa2020473

[14] Schucht P Fischer U Fung C Follow-up computed tomography after evacuation of chronic subdural hematoma. N Engl J Med. 2019;380(12):1186-1187. 30893542 10.1056/NEJMc1812507

[15] Liu J Ni W Zuo Q Middle meningeal artery embolization for nonacute subdural hematoma. N Engl J Med. 2024;391(20):1901-1912. 39565989 10.1056/NEJMoa2401201

[16] Davies JM Knopman J Mokin M Adjunctive middle meningeal artery embolization for subdural hematoma. N Engl J Med. 2024;391(20):1890-1900. 39565988 10.1056/NEJMoa2313472

[17] Fiorella D Monteith SJ Hanel R Embolization of the middle meningeal artery for chronic subdural hematoma. N Engl J Med. 2025;392(9):855-864. 39565980 10.1056/NEJMoa2409845

[18] Miah IP Holl DC Blaauw J Dexamethasone versus surgery for chronic subdural hematoma. N Engl J Med. 2023;388(24):2230-2240. 37314705 10.1056/NEJMoa2216767

[19] Penchet G Loiseau H Castel JP. Chronic bilateral subdural hematomas. Neurochirugie. 1998;44(4):247-252. 9864695

[20] Torihashi K Sadamasa N Yoshida K Narumi O Chin M Yamagata S. Independent predictors for recurrence of chronic subdural hematoma: a review of 343 consecutive surgical cases. Neurosurgery. 2008;63(6):1125-1129. 19008766 10.1227/01.NEU.0000335782.60059.17

[21] Santarius T Kirkpatrick PJ Ganesan D Use of drains versus no drains after burr-hole evacuation of chronic subdural haematoma: a randomised controlled trial. Lancet. 2009;374(9695):1067-1073. 19782872 10.1016/S0140-6736(09)61115-6

[22] Kellogg RT Vargas J Barros G Segmentation of chronic subdural hematomas using 3D convolutional neural networks. World Neurosurg. 2021;148:e58-e65. 33359736 10.1016/j.wneu.2020.12.014

[23] Vargas J Harrison Snyder M Blalock J Automated preoperative and postoperative volume estimates risk of retreatment in chronic subdural hematoma: a retrospective, multicenter study. Neurosurgery. 2024;94(2):317-324. 37747231 10.1227/neu.0000000000002667

[24] Tsutsumi K Maeda K Iijima A Usui M Okada Y Kirino T. The relationship of preoperative magnetic resonance imaging findings and closed system drainage in the recurrence of chronic subdural hematoma. J Neurosurg. 1997;87(6):870-875. 9384397 10.3171/jns.1997.87.6.0870

[25] Robinson RG. Chronic subdural hematoma: surgical management in 133 patients. J Neurosurg. 1984;61(2):263-268. 6737050 10.3171/jns.1984.61.2.0263

[26] Gelabert-González M Iglesias-Pais M García-Allut A Martínez-Rumbo R. Chronic subdural haematoma: surgical treatment and outcome in 1000 cases. Clin Neurol Neurosurg. 2005;107(3):223-229. 15823679 10.1016/j.clineuro.2004.09.015

[27] Blaauw J Jacobs B den Hertog HM Neurosurgical and perioperative management of chronic subdural hematoma. Front Neurol. 2020;11:550. 32636797 10.3389/fneur.2020.00550 PMC7317017

[28] Spallone A Giuffrè R Gagliardi FM Vagnozzi R. Chronic subdural hematoma in extremely aged patients. Eur Neurol. 1989;29(1):18-22. 2707288 10.1159/000116370

[29] Baechli H Nordmann A Bucher HC Gratzl O. Demographics and prevalent risk factors of chronic subdural haematoma: results of a large single-center cohort study. Neurosurg Rev. 2004;27(4):263-266. 15148652 10.1007/s10143-004-0337-6

[30] Lindvall P Koskinen LOD. Anticoagulants and antiplatelet agents and the risk of development and recurrence of chronic subdural haematomas. J Clin Neurosci. 2009;16(10):1287-1290. 19564115 10.1016/j.jocn.2009.01.001

[31] De Bonis P Trevisi G de Waure C Antiplatelet/anticoagulant agents and chronic subdural hematoma in the elderly. PLoS One. 2013;8(7):e68732. 23874740 10.1371/journal.pone.0068732 PMC3709887

[32] Eagle SR Mittal AM Kellogg RT Interaction of admission platelet count with current medications and the risk for chronic subdural recurrence. Neurosurg Focus. 2023;55(4):e4. 10.3171/2023.7.FOCUS23240 37778037

[33] Andersen-Ranberg NC Poulsen FR Bergholt B Hundsholt T Fugleholm K. Bilateral chronic subdural hematoma: unilateral or bilateral drainage? J Neurosurg. 2017;126(6):1905-1911. 27392267 10.3171/2016.4.JNS152642

