# Supplementary material for: Comparison of Bilateral and Unilateral Chronic Subdural Hematomas: A Retrospective Multi-institutional Cohort Study
Source: Neurosurg Pract. 2026 Jul 9;7(4):e000261. doi: 10.1227/neuprac.0000000000000261 (PMC13344927; doi:10.1227/neuprac.0000000000000261)
Supplement: Supplementary file 1 [file neuopen-7-e000261-s001.docx]

**Supplemental 1:** Baseline characteristics and outcomes of patients with unilateral cSDH, bilateral cSDH treated on one side, and bilateral cSDH treated on both sides.

| Variable | Bilateral cSDH operated on one side N = 36 | Bilateral cSDH operated on both sides N = 132 | Unilateral cSDH N = 563 | p-value |
| --- | --- | --- | --- | --- |
| Gender |  |  |  | 0.041* |
| Female | 5 (14%) | 31 (23%) | 171 (30%) |  |
| Male | 31 (86%) | 101 (77%) | 392 (70%) |  |
| Age | 69.1 ± 12.7 | 74.0 ± 10.2 | 71.5 ± 12.2 | 0.067* |
| Admission GCS | 13.5 ± 2.6 | 14.4 ± 1.4 | 14.3 ± 1.6 | 0.3 |
| Midline Shift | 6.0 ± 3.1 | 3.6 ± 3.5 | 8.5 ± 4.5 | <0.001* |
| Anticoagulation | 10 (28%) | 34 (26%) | 82 (15%) | 0.002* |
| Antiplatelet | 22 (61%) | 51 (39%) | 260 (46%) | 0.046* |
| Admission platelet count (U/mL) | 221.5 ± 52.6 | 229.1 ± 91.9 | 234.4 ± 92.0 | 0.6 |
| Preoperative cSDH Volume (mL) | 156.0 ± 57.2 | 170.2 ± 58.4 | 108.2 ± 42.4 | <0.001* |
| Postoperative cSDH Volume (mL) | 90.7 ± 41.0 | 74.1 ± 44.9 | 47.0 ± 31.4 | <0.001* |
| Length of Stay | 15.4 ± 40.8 | 7.3 ± 5.2 | 7.3 ± 7.0 | 0.10 |
| Thirty Day Readmission | 5 (14%) | 40 (30%) | 143 (25%) | 0.13 |
| Thirty Day Mortality | 3 (8.3%) | 7 (5.3%) | 45 (8.0%) | 0.6 |
| Retreatment | 4 (11%) | 19 (14%) | 65 (12%) | 0.7 |
| Retreatment time | 26.3 ± 19.3 | 31.4 ± 20.0 | 16.3 ± 18.0 | 0.002* |
| Chronic Subdural Hematoma (cSDH); Glasgow Coma Scale (GCS)  Categorical variables are displayed as n (%); Continuous variables are displayed as Mean ± SD  Pearson’s Chi-squared test, Kruskal-Wallis rank sum test, and Fisher’s exact test were used as appropriate based on variable type and distribution  *Statistically significant values | | | | |
